# Osteoid Osteoma of the Coracoid Process Presenting as Adhesive Capsulitis in a 10-Year-Old Male: A Case Report

**DOI:** 10.1055/s-0042-1751241

**Published:** 2022-07-27

**Authors:** Anthony Mancuso, Amy Singleton, Hadeel Abaza, Michael Albert, Jeffrey Mikutis

**Affiliations:** 1 Department of Orthopaedic Surgery, Mercy Health – Saint Vincent Medical Center, Toledo, Ohio, United States; 2 Department of Orthopaedic Surgery, Dayton Children's Hospital, Dayton, Ohio, United States

**Keywords:** osteoid osteoma, coracoid process, adhesive capsulitis, primary bone tumor, benign bony lesion

## Abstract

A 10-year-old male presented with symptoms in his right shoulder indicative of adhesive capsulitis. Radiographic films did not demonstrate any osseous abnormalities. Magnetic resonance imaging demonstrated the presence of an eccentric lesion within the coracoid process consistent with an osteoid osteoma. Six months after surgical removal the patient is back to full activities. For the pediatric population, surgeons must always consider diagnoses that could alter a patient's growth or result in long-term disability. In particular, an atypical presentation of musculoskeletal disease in a pediatric patient presenting with a disease that typically is seen in the older population warrants further workup.

## Introduction

Osteoid osteomas are small, benign bony lesions accounting for 3% of all primary bone tumors that are frequently diagnosed in younger patients, and in particular the male population. 1 Approximately 80% of all osteoid osteomas are diagnosed in individuals under the age of 30. Typically, osteoid osteomas are diagnosed at < 2 cm in size and affect the diaphyseal portion of long bones, commonly the femur or tibia. Osteoid osteomas can also be found in the spine, upper extremity, hands, or feet, and rarely in flat bones such as the skull or scapula. 2 3 Discussion of treatment of these lesions found in these rare locations are limited to case reports in the literature.

Characteristically, osteoid osteomas present with generalized pain in the region of the benign tumor which patients often state to be more frequent and worse at night; the classic sign of nyctalgia. 1 4 Fortunately, the pain is usually successfully treated with anti-inflammatory medications such as ibuprofen. For cases where the patient and their family can no longer tolerate the pain, or if there are complications associated with the osteoid osteoma, such as growth disturbances, changes in their quality of life, worsening scoliosis, or development of osteoarthrosis, operative treatment is warranted. Operative treatment for removal of osteoid osteomas is usually successful and includes open excision, percutaneous drilling, cryoablation, and radiofrequency ablation. 5 6 7 8

In contrast, adhesive capsulitis or “frozen shoulder,” is a common diagnosis of the glenohumeral joint occurring in patients typically 40 to 60 years of age. Very rarely is frozen shoulder diagnosed in the pediatric population. 9 Radiographs are typically normal with physical exam leading to a diagnosis. There is an equal loss of both active and passive range of motion, with the most common physical exam finding being loss of passive external rotation. Treatment is typically conservative with physical therapy, but if conservative treatment fails surgical treatments are available.

There have been reports of shoulder pain caused by neoplasms, but rarely do the patients present with frozen shoulder symptoms. Often complaints are related to bursitis, impingement, or instability. 10 11 This case report discusses a 10-year-old boy presenting to the orthopaedic clinic with many months of atraumatic generalized right shoulder pain and weakness especially with baseball activities. Interestingly, our patient also presented with the classic signs and symptoms of adhesive capsulitis, a rare diagnosis in the pediatric population.

## Case Presentation

A 10-year-old male patient presented to our clinic due to increasing weakness, vague pain, and global stiffness over many months in his right shoulder. The patient was noted to be ambidextrous with sporting activities and began to notice increasing right arm weakness compared with the left while playing baseball. He denied numbness or tingling to the right upper extremity and described the pain as a pressure or similar to a “toothache.” He denied any history of trauma to the right upper extremity.

**Fig. 1:**
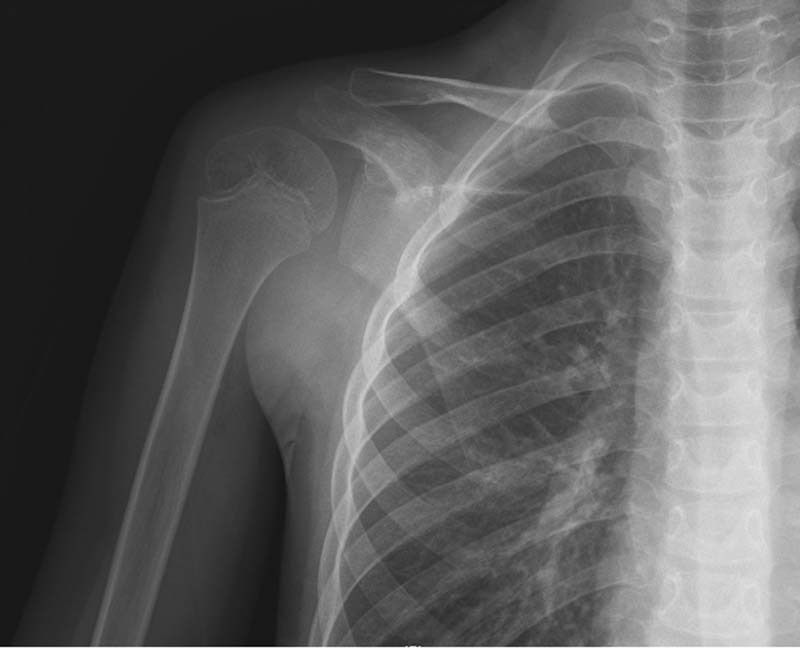
Anteroposterior (AP) X-ray of the right shoulder at initial presentation demonstrating no obvious deformity, dislocation, or malignancy.

Physical exam demonstrated significantly decreased muscle strength grade (a full grade lower) and range of motion (by at least 50%) of the right shoulder in all planes (flexion, abduction, adduction, internal, and external rotation) compared with the left. No appreciable point of tenderness, mass, or overt signs of infection were seen, and he was neurovascularly intact. Plain radiographic films did not demonstrate any appreciable fracture, dislocation, or osseous abnormalities of the right upper extremity ( Fig. 1 ). Given the atypical presentation of the patient's right shoulder pain, weakness, and decreased range of motion further workup was needed to identify a cause.

**Fig. 2:**
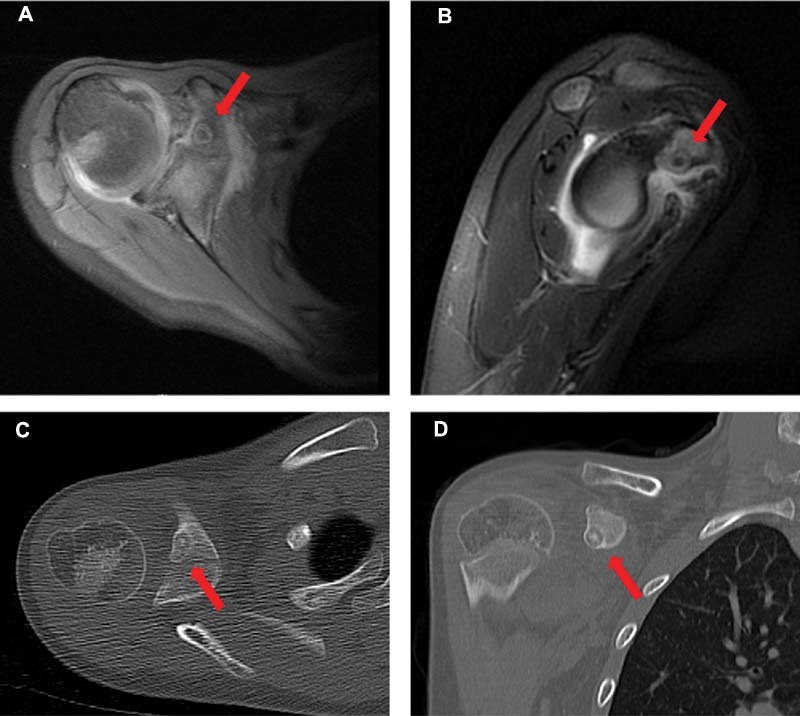
T2-weighted magnetic resonance imaging (MRI) and computed tomography (CT) images of the right shoulder demonstrating an osteoid osteoma. Red arrows point to location of lesion. ( **A** ) Axial MRI image. ( **B** ) Sagittal MRI image. ( **C** ) Axial CT image. ( **D** ) Sagittal CT image.

The patient's laboratory evaluation was normal with no signs of infection; rheumatologic and Lyme disease workup were also negative. Magnetic resonance imaging (MRI) of the right shoulder demonstrated the presence of an eccentric lesion within the coracoid process; further characterized with computerized tomography (CT) ( Fig. 2 ) and consistent with an osteoid osteoma.

The patient and his family opted for surgical treatment given the patient's continued discomfort and limitations in activities. A CT was obtained for preoperative planning. On the day of surgery, general anesthesia was induced, and the patient was taken for CT-guided, percutaneous guide pin placement directly within the bony lesion. Once in the operating room, the guide pin was used to help localize the lesion guiding its removal with curettage. Given the lack of soft tissue mass or concerning malignant findings we felt that having pathologic evaluation during surgery was not needed for confirmation. A biopsy was taken and pathologic investigation of the lesion postoperation confirmed the diagnosis of osteoid osteoma. At 6 months postoperation, the patient had regained range of motion and strength of his right shoulder to be equivalent with his left shoulder. He was back to full activities, including baseball, without restriction and had no further complaints of shoulder pain. The patient and his legal guardian have given consent for this case report to be published.

## Discussion

Osteoid osteomas are benign lesions usually found in the lower extremities of pediatric patients, although upper extremity osteoid osteomas do still occur. 4 There have been multiple case reports of patients with osteoid osteomas presenting to the clinic with complaints and classic symptoms of other musculoskeletal diseases from thoracic outlet syndrome to wrist arthritis leading to delayed diagnosis and treatment. 7 12 13 14 Patients with osteoid osteomas also typically present with the classic complaint of night pain that is alleviated by anti-inflammatory medications. The inflammatory process associated with osteoid osteomas can certainly lead to musculoskeletal symptoms and even adhesive capsulitis symptoms as seen in this patient. Adhesive capsulitis in the pediatric population is rare and should prompt consideration of other etiologies for the patient's pain and stiffness. Removal of the osteoid osteoma halts the inflammatory process as seen in this patient where he had a full recovery and was symptom free at 6 months postoperation.

Given the rarity of presentation of adhesive capsulitis in the pediatric population a workup must include screening for infectious and inflammatory etiologies. This should also include evaluation for benign and/or malignant lesions with appropriate laboratory evaluation and advanced imaging as justified. 10 11 15 For uncommon presentations in the pediatric population, a physical exam is insufficient to either rule in or out a diagnosis. Interestingly, in a study that explored frozen shoulder syndromes in 50 patients and found that it was caused by shoulder girdle neoplasms in 7 patients, in all 7 of the patients, a point of discrete tenderness was present, which was not present in our patient. 16 Physical exam must be paired appropriately with further workup. Plain film X-ray imaging is insufficient to characterize and localize the lesion, and MRI along with CT are often utilized. 1 16 A discussion of the case in an interdisciplinary tumor board is also beneficial if institutionally available.

The danger in delaying proper diagnosis is the potential for causing iatrogenic harm. For this case, potential treatment for his refractory frozen shoulder could have included manipulation under anesthesia or arthroscopy which would have unnecessarily exposed him to the risks of anesthesia and to an invasive procedure. 16

## Conclusion

For this patient, successful diagnosis and treatment of the osteoid osteoma resulted in excellent outcomes and iatrogenic harm was further avoided. For the pediatric population, surgeons must always consider diagnoses that could alter a patient's growth or result in long-term disability, and even more so with atypical presentations of musculoskeletal diseases such as in this case with a pediatric patient presenting with a disease that typically is seen in the older population. In pediatric cases such as this one, the appropriate imaging, physical exams, and differential building will need to be adjusted from what would most often be considered in an adult case. Further work into guidelines for appropriate diagnostic steps is needed.

## Notes

New Insights and Importance for the Pediatric Surgeon For the pediatric population, surgeons must always consider diagnoses that could alter a patient's growth or result in long term disability. In particular, an atypical presentation of musculoskeletal disease in a pediatric patient presenting with a disease that typically is seen in the older population warrants further workup. Appropriate imaging, physical exams, and differential building will need adjustment from what would most often be considered in an adult case. Further work into guidelines for appropriate diagnostic steps is needed.
